# Adaptation and Validation of Portuguese Version of Olfactory Disorders Questionnaire (PT-ODQ)

**DOI:** 10.7759/cureus.29939

**Published:** 2022-10-05

**Authors:** André De Sousa Machado, Francisco Sousa, Joana Costa, Ana Silva, Ana Pinto, Daniel Simmen, Hans R Briner, Luis Meireles

**Affiliations:** 1 Otolaryngology, University Hospital Center of Porto (CHUPorto), Porto, PRT; 2 Medical Education and Simulation, University of Beira Interior, Covilha, PRT; 3 Otolaryngology, Hirslanden Private Hospital Group, Zurich, CHE

**Keywords:** portuguese, validation study, questionnaire study, hyposmia, olfactory

## Abstract

Introduction

Olfactory dysfunction (OD) is often a devaluated sensorial affection. The objective evaluation of this dysfunction doesn’t evaluate its compromise in patients’ daily life. Therefore, the use of a Portuguese-validated tool is of uttermost importance to objectively scale the pathology presented by these patients.

Objective

We aim to validate and cross-culturally adapt the Olfactory Disorders Questionnaire (ODQ) in the Portuguese language.

Methods

A prospective study was carried out to evaluate and compare 56 consecutive patients who had olfactory disorders and 54 asymptomatic controls. A cross-cultural adaptation process was taken into account in order to transform the original English tool into a valid Portuguese version. We explored the psychometric properties of the European-Portuguese version of the Portuguese version of ODQ (PT-ODQ) concerning its internal consistency, reproducibility, feasibility, and discriminatory validity.

Results

Cronbach alpha for the tool was 0.924 showing strong internal consistency. We also found a statistically significant difference in PT-ODQ between patients with olfactory disorders and patients without olfactory disorders, according to the Mann-Whitney test.

Conclusions

The PT-ODQ seems to be a valid tool for assessing the individual effect of olfactory disorders on patients’ quality of life and, therefore, could be applied in olfactory disorders research and daily practice.

## Introduction

Olfaction is fundamental for appetite, elicits avoidance of environmental hazards, and ultimately influences social connections and well-being [1]. Compared with other sensorial affections, a reduced olfactory function is commonly undiagnosed or devalued [1]. On the other hand, in some cases, the olfactory impairment may imply important psychological consequences [2-5]. Olfactory dysfunction (OD) is often a devaluated sensorial affection, and those who suffer from it can feel misunderstood and hopeless [6]. The COVID-19 pandemic shed light on the importance of OD since a significant number of infected people worldwide suffered from transient or long-lasting OD after SARS-CoV-2 infection [7-11]. The authors of this article developed a targeted consultation for post-COVID-19 persistent OD after realizing that there were no specific and organized medical responses to the post-COVID-19 persistent OD throughout the pandemic.

In the consultation, objective evaluation of OD, by means of threshold, discrimination, and identification (TDI score), allows clinicians to confirm the existence of OD; however, it does not let one determine the impact of OD and associated symptoms on the patient's quality of life. Thus, there was a need to use other methods to make this assessment in the most targeted way. There are validated questionnaires for English and other languages, such as the English Olfactory Disorders Questionnaire (ODQ) by Langstaff et al. [12], which was adapted from the original Questionnaire of Olfactory Disorders created by Frasnelli et al. in Germany [13]. This was the first questionnaire that specifically addressed OD and its daily life impact, rating several domains of daily life [13]. Several studies have now translated or used this questionnaire [14-17]. Nevertheless, the authors did not find any similar olfactory disorders questionnaire validated for the Portuguese language that could be used.

Hence, the purpose of this work was to validate the Portuguese version of the Questionnaire of Olfactory Disorders (PT-ODQ) in order to be applied to Portuguese speakers and allow clinicians to access the impact of OD on patients' quality of life (QoL).

## Materials and methods

Design

This prospective study was performed at the Department of Otolaryngology of a tertiary center, University Hospital Center of Porto, between April 2020 and February 2021.

Ethics

Institutional ethical approval was obtained prior to the study, and the design is in line with Helsinki Declaration. Formal consent was signed by the enrolled patients after the description of the study.

Inclusion criteria

To validate the PT-ODQ, both case and control groups were enrolled. The case group included patients suffering from objective and subjective post-infectious persistent olfactory dysfunction, whereas the control group included normal controls without olfactory complaints. For the case group, inclusion criteria were age ≥18 years, subjective persistence of olfactory dysfunction, and cognitive ability to sign informed consent. For the control group, inclusion criteria were age ≥ 18 years, no prior known nasosinusal pathology, cognitive ability to sign an informed consent, and normosmia documented by Sniffin Sticks® (Burghardt, Wedel, Germany).

Exclusion criteria

For both groups, exclusion criteria excluded patients with recent head trauma with loss of consciousness, previously documented olfactory dysfunction, pregnancy, former neurological or nasal surgery, and patients who presented with a neurologic or psychiatric disease previously diagnosed.

Variables evaluated

For each group, age and gender were taken into account. The olfactory threshold was evaluated with Sniffin Sticks® (0 to 16) in each patient. Also, the total score of PT-ODQ (sum of the score in each question), minimum, and maximum values were assessed in each patient.

Translation

After obtaining permission from the authors of the E-ODQ [12], the English to European-Portuguese translation was done separately by two native-speaking translators with medical knowledge, following guidelines of forward and backward translation. The two versions were combined into onw edition (Table 1) and then reviewed by the otolaryngologists. The questionnaire, originally with 31 questions, was adapted to a Portuguese version of 17 questions. To ensure understandability, the Portuguese version was then distributed to 15 individuals whose feedback was considered, with small detail refinement, after which the final questionnaire was obtained.

**Table 1:** Portuguese language translation of the questionnaire Each response was rated on a scale of 0 to 3, with 0 indicating strong disagreement, 1 indicating partial disagreement, 2 indicating partial agreement, and 3 indicating strong agreement with the statement.

Regarding your sense of smell and taste, answer the following questions:	Relativamente ao seu olfacto e paladar, responda às seguintes questões:
Q1. Because of changes in my sense of smell, I go out less often than I used to.	Q1. Por causa das mudanças no olfato, vou a restaurantes com menos frequência do que costumava.
Q2. I am always aware of changes in my sense of smell.	Q2. Estou sempre ciente das mudanças no meu olfato.
Q3. Because of changes in my sense of smell, I don't like drinks or food as much as I used to.	Q3. Por causa das mudanças no meu olfato, não gosto de bebidas ou comida tanto quanto costumava gostar.
Q4. I'm worried I'll never get used to the changes in my sense of smell.	Q4. Estou preocupado que nunca vou me acostumar com as mudanças no meu olfato.
Q5. Because of the changes in mine, I feel more anxious than I used to.	Q5. Por causa das mudanças no meu olfato, sinto-me mais ansioso do que me costumava sentir.
Q6. Changes in my sense of smell cause most of my problems.	Q6. As mudanças no meu olfato causam a maioria dos meus problemas.
Q7. Changes in my sense of smell bother me when I'm eating.	Q7. As mudanças no meu olfato incomodam-me quando estou a comer.
Q8. Because of changes in my sense of smell, I visit friends, relatives, or neighbors less often.	Q8. Por causa das mudanças no meu olfato, visito amigos, parentes ou vizinhos com menos frequência.
Q9. Because of the changes in my sense of smell, it's harder for me to relax.	Q9. Por causa das mudanças no meu olfato, eu esforço-me mais para conseguir relaxar.
Q10. Because of the changes in my sense of smell, I have weight problems.	Q10. Por causa das mudanças no meu olfato, tenho problemas de peso.
Q11. Changes in my sense of smell make me feel isolated.	Q11. As mudanças no meu olfato fazem-me sentir isolado.
Q12. Because of changes in my sense of smell, I avoid groups of people.	Q12. Por causa das mudanças no meu olfato, evito grupos de pessoas.
Q13. Because of changes in my sense of smell, I eat less than I used to or more than I used to.	Q13. Por causa das mudanças no meu olfato, como menos do que costumava ou mais do que costumava.
Q14. Because of difficulties with my sense of smell, I am afraid of being exposed to certain dangers (e.g. gas, spoiled food).	Q14. Por causa das dificuldades com o olfato, tenho medo de ficar exposto a certos perigos (por exemplo, gases, comida estragada).
Q15. Because of changes in my sense of smell, I have trouble participating in activities of daily living.	Q15. Por causa das mudanças no meu olfato, tenho problemas em participar em atividades da vida diária.
Q16. The changes in my sense of smell make me feel angry.	Q16. As mudanças no meu olfato fazem-me sentir raiva.
Q17. Because of the changes in smell, the relationship with my partner is affected.	Q17. Por causa das mudanças no meu olfato, o relacionamento com minha esposa/marido/companheiro é afetado.

Statistical analysis

The data were analyzed using the Statistical Package for Social Studies version 27 (IBM Inc., Armonk, New York). For continuous variables, mean, standard deviation, and range were calculated. The differences between the means were obtained using the Mann-Whitney U test.

The correlation between questions was done using Spearman's correlation test. The correlation between the total score and each domain of PT-ODQ was performed with Pearson's correlation test.

To evaluate the test-retest reliability of the PT-ODQ, the ques­tionnaire was administered twice approximately two weeks apart to all patients - no change was expected to occur in olfactory function in such time but being sufficient to avoid recall biases while answering. When completing the second PT-ODQ, the subjects were not allowed to check their responses to the previous question­naire.

## Results

Fulfillment time

All patients included filled the questionnaire in a short time (4.1 ± 1.2 min).

Demographic characteristics 

Our sample was composed of 110 patients (53 females, 57 males). Fifty-six patients with olfactory dysfunction composed the case group (1) (50.9%) with olfactory thresholds of 4.68±2.241. Fifty-four patients composed the control group (2) (49.1%) with olfactory thresholds of 14.67±0.752 (Table 2).

**Table 2:** Demographic variables PT-ODQ - Portuguese version of the Questionnaire of Olfactory Disorders; OD - olfactory disorder; SD - standard deviation

Variables	Group 1 (OD group)	Group 2 (Control group)
Number (n)	56	54
Age in years (±SD)	37.16±11.414	42.33±3.120
Males	21	36
Females	35	18
Olfactory threshold (±SD)	4.68±2.241	14.67±0.752
PT-ODQ mean value	20.196±10.283	1.92±3.08
PT-ODQ minimum value	3	0
PT-ODQ maximum value	44	8

The comparison between patients with hyposmia (first group) and the control group was assessed with the Mann-Whitney U test. The total score of PT-ODQ (sum of the score in each question) in the first group ranged between three and 44 with a mean value of 20.196 ± 10.283, while in the control group, the total score ranged between 0 and eight with a mean of 1.92 ± 3.08. There was a statistically significant difference between the two groups (p-value=0.001) (Table 2).

Reliability

The internal consistency was assessed by Cronbach's alpha - this value was 0.924 (very high). Test-retest reliability for the Portuguese version was confirmed by the Pearson correlation coefficient (r=0.91, N=56, p<0.001). A significant test-retest reliability was also found between the Portuguese and the English version (r=0.9, N=12, p<0.001). All items showed a Pearson's r>0.70 in the test-retest reliability analysis (Table 3).

**Table 3:** Validation tests of the PT-ODQ PT-ODQ: Portuguese Olfactory Disorders Questionnaire

Questions of the delivered questionnaire and respective statistic analysis	r	p-value
Test-retest correlation		
Q1	0.87	<0.001
Q2	0.88	<0.001
Q3	0.82	<0.001
Q4	0.77	0.02
Q5	0.90	<0.001
Q6	0.92	<0.001
Q7	0.75	<0.001
Q8	0.75	<0.001
Q9	0.86	<0.001
Q10	0.79	<0.001
Q11	0.91	<0.001
Q12	0.87	<0.001
Q13	0.88	<0.001
Q14	0.82	<0.001
Q15	0.77	0.02
Q16	0.90	<0.001
Q17	0.83	<0.001
Total	0.91	<0.001
Portuguese-English test-retest correlation		
Q1	0.72	0.03
Q2	0.79	0.03
Q3	0.82	<0.001
Q4	0.95	<0.001
Q5	0.94	<0.001
Q6	0.98	<0.001
Q7	0.91	<0.001
Q8	0.82	<0.001
Q9	0.95	<0.001
Q10	0.94	<0.001
Q11	0.98	<0.001
Q12	0.91	<0.001
Q13	0.82	<0.001
Q14	0.95	<0.001
Q15	0.94	<0.001
Q16	0.98	<0.001
Q17	0.91	<0.001
Total	0.90	<0.001

## Discussion

Olfaction is an intriguing sense and plays a major role in health, social interaction, and self-protection from harmful stimuli, therefore having a considerable impact on quality of life [1]. Since olfaction relates closely with emotional status, some studies have shown a connection between olfaction and depressive disorders [1, 18].

The goal of this study was to adapt and validate the questionnaire into the Portuguese language.

The presented translation was obtained by following the rules of translation, producing a robust, easily and clearly understood version completed by all the participants [19]. The short time of fulfillment of all items without any help confirms the feasibility of our version [20].

Reproducibility is the ability to provide similar results when the patient's status does not alter through time - the reliability coefficient indicates a stable reproducible version. The strength of a research tool is linked to internal consistency. In this study, Cronbach's alpha was 0.946, making this version consistent [20].

Validity is the ability of a questionnaire to successfully measure its target. All correlations in the translated questionnaire obtained a positive correlation with the original questionnaire, and by so, confirming the validity of the translated questionnaire [20].

Our research tool proved to be responsive, detecting changes over time, confirmed by the comparison between the total score and subscale scores during the test-retes with significant outcomes [19]. Previously published scores are in line with those observed in this study, confirming that the shortness of the questionnaire does not lessen its ability to discriminate pathologies [14, 19].

As a limitation of our study, we point out that our sample didn't consider patients with conductive olfactory loss, such as chronic rhinosinusitis - a condition that can present with hyposmia. Although we believe that our findings aren't affected by this, as the main referral of these patients was, in fact, olfactory dysfunction. The size of the sample can also be pointed out as a limitation.

Also, the role of several therapeutics in the olfactory dysfunction group might be of great interest to evaluate changes with intervention. Further studies must be enrolled in order to evaluate this issue.

## Conclusions

As one of the COVID-19 sequelae problems is olfactory dysfunction, people have become more aware of this feature. We believe the use of this tool is of great interest for the physician in order to stratify and categorize the population that presents such complaints, and we also believe the presentation of this questionnaire might go in line with patients' complaints that can feel misunderstood and neglected.

According to our results, PT-ODQ is a valid tool and a strong subjective method to detect the effects of olfaction impairment on the health and quality of life of patients and, therefore, has a role in the clinical practice of patients who present with complaints of olfactory dysfunction.
